# Application of Organo-Magadiites for the Removal of Eosin Dye from Aqueous Solutions: Thermal Treatment and Regeneration

**DOI:** 10.3390/molecules23092280

**Published:** 2018-09-06

**Authors:** Fethi Kooli, Yan Liu, Mostafa Abboudi, Souad Rakass, Hicham Oudghiri Hassani, Sheikh Muhammad Ibrahim, Rawan Al-Faze

**Affiliations:** 1 Community College, Taibah University-Al-Mahd Branch, Al-Mahd 42112, Saudi Arabia; 2 Institute of Chemical and Engineering Sciences, 1 Pesek Road, Jurong Island, Singapore 627833, Singapore; liu_yan@ices.a-star.edu.sg; 3 Department of Chemistry, Taibah University, P.O. Box 30002, Al-Madinah Al-Munawwarah 41147, Saudi Arabia; abboudi14@hotmail.com (M.A.); rakass_souad@yahoo.fr (S.R.); oudghiri_hassani_hicham@yahoo.com (H.O.H.); mdibrahi@gmail.com (S.M.I.); rawan.faze@gmail.com (R.A.-F.); 4 Département de Chimie, Faculté des Sciences Dhar El Mahraz, Université Sidi Mohamed Ben Abdellah, B. P. 1796 (Atlas), Fès 30003, Morocco

**Keywords:** magadiite, organo-layered silicate, removal, eosin, thermal stability, regeneration

## Abstract

Na-magadiite exchanged with cetyl-trimethylammonium cations provided organophilic silicate materials that allowed for the effective removal of the acidic dye “eosin”. The organic cations were intercalated into the interlayer spacing of the layered silicate via an exchange reaction between the organic cations from their bromide salt and the solid Na-magadiite at room temperature. Different techniques were used to characterize the effect of the initial concentration of the surfactant on the structure of the organo-magadiites. The C, H, and N analysis indicated that a maximum of organic cations of 0.97 mmol/g was achieved and was accompanied by an expansion of the basal spacing of 3.08 nm, with a tilted angle of 59° to the silicate layers. The conformation of the organic surfactants was probed using solid-state ^13^C, finding mainly the *trans* conformation similar to that of the starting cetyl trimethylammonium bromide salt (C16TMABr). Thermal gravimetric analysis was carried out to study the thermal stability of the resulting organo-magadiites. The intercalated surfactants started to decompose at 200 °C, with a mass loss percentage of 8% to 25%, depending on the initial loading of the surfactant, and was accompanied by a decrease of the basal spacing from 3.16 nm to 2.51 nm, as deduced from the in situ X-ray diffraction studies. At temperatures below 220 °C, an expansion of the basal spacing from 3.15 to 3.34 nm occurred. These materials were used as a removal agent for the anionic dye eosin. The maximum amount of the dye removed was related to the organic cation content and to the initial concentration of eosin, with an improvement from 2.5 mg/g to 80.65 mg/g. This value decreased when the organo-magadiite was preheated at temperatures above 200 °C. The regeneration tests indicated that an 85% removal efficiency was maintained after six cycles of use for the organo-magadiite using C_i_ of 200 mg/L.

## 1. Introduction

Layered materials have attracted great attention due to their wide application in different fields and due to their characteristic cation exchange capacity, acceptance of intercalated guest molecules, exfoliation in different polymer matrixes, and catalytic properties [1]. The layered silicate, magadiite, is an important member of this family, and it is easily prepared in the laboratory by the hydrothermal treatment of a silica source dissolved in an alkaline solution at a specific temperature of 150 °C for a period of time varying from two to three days [2]. Magadiite consists of multiple negatively charged layers of tetrahedral SiO_4_ with abundant silanol groups on their surfaces. The negative charge is balanced by exchangeable hydrated cations, such as Na^+^ or protons (H^+^), in the interlayer spacing [3]. The magadiite exhibited a theoretical high cation exchange capacity (CEC) of nearly 200 meq/100 g [4], resulting in good ion exchange properties as a host for many organic and inorganic cations [5,6,7,8,9,10,11]. Magadiite was used as a silica precursor to prepare some new microporous zeolite materials with unique structures and properties using small organic templates [12,13]. The acidic properties of the magadiite could be enhanced by the insertion of other cations into the layered silicate, achieved during the synthesis by adding the cations precursors to the mixture of the silica source and NaOH. This adjustment allowed for the diversification of magadiite as a catalyst in special catalytic reactions [14,15,16]. However, using long alkyl ammonium cations, the modified cetyltrimethylammonium-magadiite was used as an intermediate precursor to synthesize mesoporous materials with a controlled distribution of pores sizes [17,18]. In the removal of dyes process, it was underlined that the removal efficiency of organo-clay minerals was affected by the surfactant alkyl chain length [19,20,21,22]. A clay mineral modified with a long-chain surfactant exhibited a higher removal capacity than did short-chain surfactant. For this purpose, cetyl trimethyl ammonium (C16TMA) was the most commonly used cation, obtained from the corresponding salt; the bromide form was used to prepare organo-clays or silicates [23,24]. The effect of the counteranion anion of surfactants was reported in a few studies [25]. In the case of organo-clays, the highest intercalated amounts were achieved using the bromide form [25], while using acid-activated clays, the C16TMAOH starting solution led to the highest uptake value [26]. In the case of a layered silicate, such as magadiite, a previous study reported that the maximum amount of intercalated organic surfactants was achieved using a C16TMAOH solution [27,28]. On the other hand, H-magadiite had difficulty modifying either a C16TMABr or C16TMACl solution [28]. 

In this study, Na-magadiite was modified by different initial C16TMABr solutions at room temperature. The chemical stability of the intercalated surfactants was investigated in NaCl, HCl, and NaOH solutions. In situ XRD technique was used to study the thermal stability of the organo-magadiites. Na-magadiite and its protonated form were used as removal agents for basic dyes, such as methylene blue and other dyes, without modification [29,30,31,32]. However, for acidic dyes, no studies were reported in the literature. Eosin Y, a heterocyclic dye containing bromine atoms, was selected as a model dye, as it is used in printing, dyeing, printing ink, and fluorescent pigments, as well as in the leather and paint industries [33] The modified magadiites and the preheat-treated organo-magadiite were applied as a removal agent for the acidic dye eosin from artificially polluted water. Different factors were investigated during the removal process of the eosin dye. The regeneration process determines the feasibility of using an applied material in large-scale operations. The resulting spent materials were regenerated by a process friendly to the environment, and their reuse was studied after different consecutive cycles. 

## 2. Materials and Characterization

### 2.1. Chemicals

**Figure 1:**
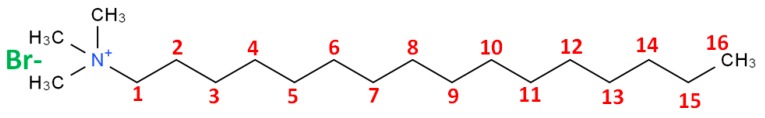
The chemical structure of the C16TMABr salt.

**Figure 2:**
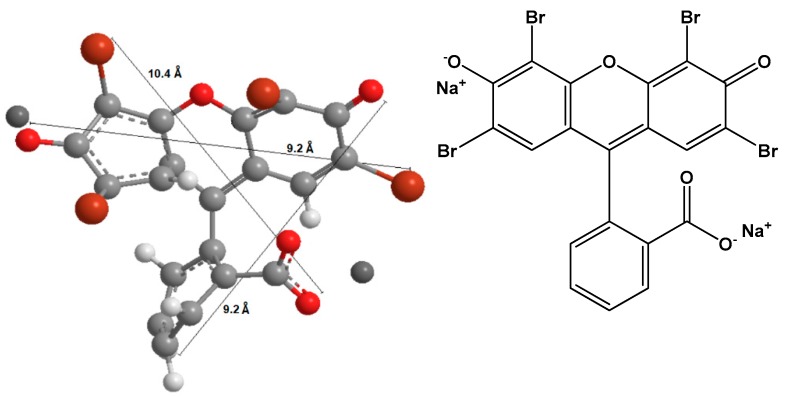
The chemical (**right**) and the optimized three-dimensional structure (**left**) of eosin Y.

We obtained fumed silica and the salt of cetyl trimethyl ammonium bromide (C16TMABr), which is a cationic surfactant with an average molar mass of 384.44 g/mol from Sigma-Aldrich (St. Louis, MO, USA). The dye eosin Y was an analytical reagent purchased from Acros Organics (Loughborough, UK). Eosin yellow, also known as Acid Red 87, C.I. 45,380 with molecular formula C_2_OH_6_Br_4_Na_2_O_5_, and molecular weight equal 691.58 g/mol. All the reagents were used as received. Figure 1 presents the chemical structure of C16TMABr salt, and Figure 2 depicts the optimized three-dimensional structural formula of the eosin Y, obtained from MarvinSketch software, version 14.9.22.0 (ChemAxon, Budapest, Hungary).

### 2.2. Na-Magadiite

Na-magadiite was prepared as reported previously [27]. Fumed silica (16 g) was added to a basic solution formed from 4.8 g of NaOH dissolved in 105 g of water in a SiO_2_/alkali ratio of 4. The mixture was sealed in a Teflon-lined autoclave and left at 150 °C for three days in a dry oven. After cooling, the product was filtered, washed with deionized water, and dried overnight in an oven at 40 °C. The Na-magadiite was named Na-mag.

### 2.3. Organo-Magadiites

Different quantities of the solid C16TMABr salt were dissolved in 50 mL of deionized water. Then, 2 g of Na-mag were added to these solutions, which were mixed at room temperature overnight. The samples were filtrated and washed with water several times (seven to eight times). The resulting materials were dried at room temperature. The samples were identified as C16Mag-X, where X represents the initial loading of the C16TMA cation in mmol per 100 g of Na-magadiite. C16Mag-40 corresponds to an organo-magadiite with a loaded amount of 40 mmol of C16TMA cations per 100 g.

### 2.4. Chemical Properties

One selected organo-magadiite (C16Mag-80) was treated with solutions of NaOH, NaCl, and HCl to explore the stability of the intercalated surfactants. For this purpose, one gram of the C16Mag-80 sample was added to 50 mL of a NaOH, HCl, or NaCl (0.5 M) solution and left overnight. The sample was filtered and washed extensively with deionised water and dried at room temperature. 

### 2.5. Eosin Removal

In the removal experiments, 100 mg of magadiite or organo-derivatives was added to a fixed volume (10 mL) of eosin dye at different initial concentrations varying from 50 mg/L to 1000 mg/L in a sealed tube. Then, the tubes were shaken laterally in a water-bath shaker at a controlled temperature of 25 °C for eighteen hours. After the separation of the two phases (solid and solution) by centrifugation, the change in the eosin concentration solution was determined by a UV spectrophotometer (Cary 100 Conc, Varian, Australia) at the maximum wavelength absorption (λ_max_) of 610 nm, using a prepared calibration curve. The experiments were carried out using duplicate samples, and the presented values are the average with an error percentage in the range of 4% to 6%.

The removal properties of a selected organo-magadiite (C16Mag-80) preheated at different temperatures were also investigated using the same procedure described above. 

### 2.6. Regeneration Studies of Spent Organo-Magadiites

The following method was applied in previous studies [34]. It is friendly to the environment since small amounts of chemicals and water solutions are used. The spent organo-magadiites were dispersed into a mixture of 10 mL of aqueous solution of Co(NO_3_)_2_·6H_2_O, and a specific amount of oxone. The mixture of the used organo-magadiite and above aqueous solution was stirred for 30 min. The solid was separated by centrifugation, washed six to seven times with deionized water, and reused for the next run. 

### 2.7. Characterization

The C, H, and N contents in the modified magadiites were carried out by an CHNS-O Analyzer from EURO EA (Waltham, MA, USA). The success of the modification of the organo-magadiites was examined using X-ray powder diffraction with a Bruker Advance 8 diffractometer (Ni-filtered Cu-Kα radiation with a wavelength of 0.154 nm, Germany) was used to obtain the XRD patterns. A field scanning electron microscopy (FSEM model JSM-6700F technique, Jeol, Japan) was performed to examine the changes in morphology of the synthesized samples. The nitrogen adsorption isotherms were performed to estimate the microtextural properties of the samples. The isotherms were measured on a Micrometrics ASAP 2040 (Ottawa, ON, Canada). Prior to the adsorption, the samples were evacuated overnight at 373 K. The surface area was estimated using the Brunauer-Emmett-Teller (BET) method, and the pore volume was deduced at a relative adsorption pressure (P/P_o_) of 0.95. Thermal gravimetry analysis (TGA) was used for the study of the thermal stability of the synthesized samples and to select the temperature values at which the organo-magadiites would be treated prior the removal studies. The analyses were made on a TA Instruments calorimeter (New Castle, DE, USA), model SDT2960. During the runs, the samples were heated to 800 °C (heating rate: 10 °C/min) in air. Solid-state NMR spectra were measured with a Bruker DSX 400 MHz instrument under Magic Angle Spinning (MAS) conditions in 2.5 mm ZrO_2_ rotors with a sample volume of 12 μL (rotation frequency: 20 kHz), as reported in previous studies.

## 3. Results and Discussion

### 3.1. C, H, and N Elemental Analysis

The carbon, nitrogen, and hydrogen analysis was used to quantify the organic contents in organo-clays and other organo-silicates [27,35,36,37]. The elemental analysis of the organo-magadiites is summarized in Table 1. The data revealed that the contents of carbon and nitrogen increased after modification with the surfactant, and then remained unchanged for initial concentrations greater than 0.80 mmol. The amount of intercalated C16TMA in the magadiite interlayer spaces varied from 0.17 mmol/g to 0.97 mmol/g. The later value was lower than the expected cation exchange capacity, and it indicated that the modification of the Na-magadiite occurred mainly via a cation exchange process. The electron dispersive X-ray analysis (EDX) data indicated that traces of Na cations occurred with high loadings of organic surfactants. In the case of the similarly layered structure kenyaite, only a partial exchange occurred [36]. This result was not the same for clay minerals, where the uptake amount of C16TMA largely exceeded the cation exchange capacity (CEC) values [24]. This variation was related to the different uptake mechanisms for surfactants and layered silicates. 

When the C, H, and N analysis was performed for the organo-magadiite treated with NaOH or NaCl solutions, the percentages of C and N did not vary, indicating that the organic surfactants were stable and did not exchange with the Na cations from the NaOH solution. However, the percentages of C and N decreased dramatically when the organo-magadiite was treated with the HCl solution, indicating that the organic surfactants were replaced by the protons of the acid solution, Similar data were obtained from organo-clays and organo-silicates. For the sample treated with deionized water, no change occurred.

### 3.2. X-ray Diffraction Data

**Figure 3:**
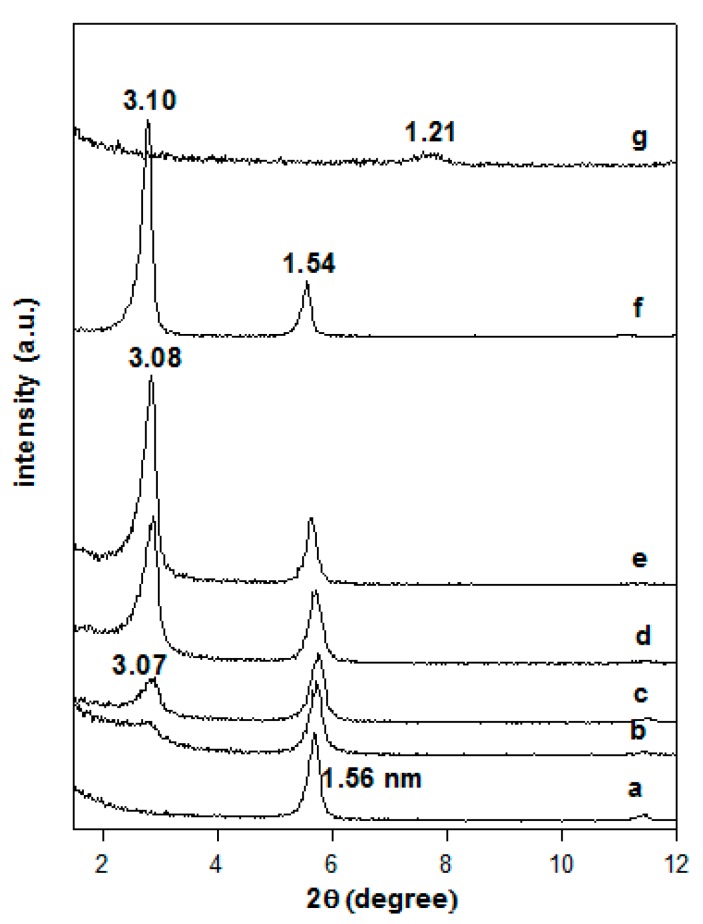
Powder XRD patterns of (**a**) Na-magadiite treated with different C16TMABr concentrations. (**b**) 0.20 mM, (**c**) 0.40 mM, (**d**) 0.80 mM, and (**e**) 1.20 mM, and (**f**) corresponds to organo-magadiite prepared from (**g**) H-magadiite and C16TMAOH solution.

The powder XRD patterns of the Na-magadiite and the organo-derivatives are presented in Figure 3. The Na-magadiite exhibited a basal spacing of 1.56 nm, close to that reported in the literature [2,38,39], when it was reacted with an initial surfactant loading of 0.40 mmol and a broad reflection corresponding to 3.07 nm was obtained, indicating that a partial exchange of the Na-magadiite occurred. The intensity of the broad reflection at 3.07 nm was improved by increasing the loading concentration of C16TMABr, and the pattern exhibited sharp reflections for a basal spacing of 3.08 nm. This value remained unchanged for initial loading concentrations greater than 0.80 mmol, while an additional reflection at 1.54 nm was detected and could be related to a second-order reflection of the first at 3.08 nm or to an unreacted Na-magadiite phase. In a previous study, a similar organo-magadiite prepared from protonated magadiite (H-mag) exhibited a first reflection at 3.10 nm and a second one at 1.54 nm. The later value was close to that of Na-magadiite and lower than the reflection of the starting protonated magadiite at 1.21 nm (Figure 3) [6,28]. These data confirmed that the reflection at 1.54 nm was indeed related to the organo-magadiite phase and not to the starting Na-magadiite phase.

The basal spacing of the organo-magadiite of 3.10 nm was higher than the value reported for organo-magadiite prepared with the same C16TMA cations (2.45 nm, [40]), This difference could be related to different arrangement of the C16TMA cations, and to the used solvent during the modification process, *N*-dimethylacetamide was employed instead of deionized water. The effect of the solvent on the arrangement of C16TMA into the basal spacing of organo-clay minerals was reported using pure ethanol or a mixture of water/ethanol [41].

**Figure 4:**
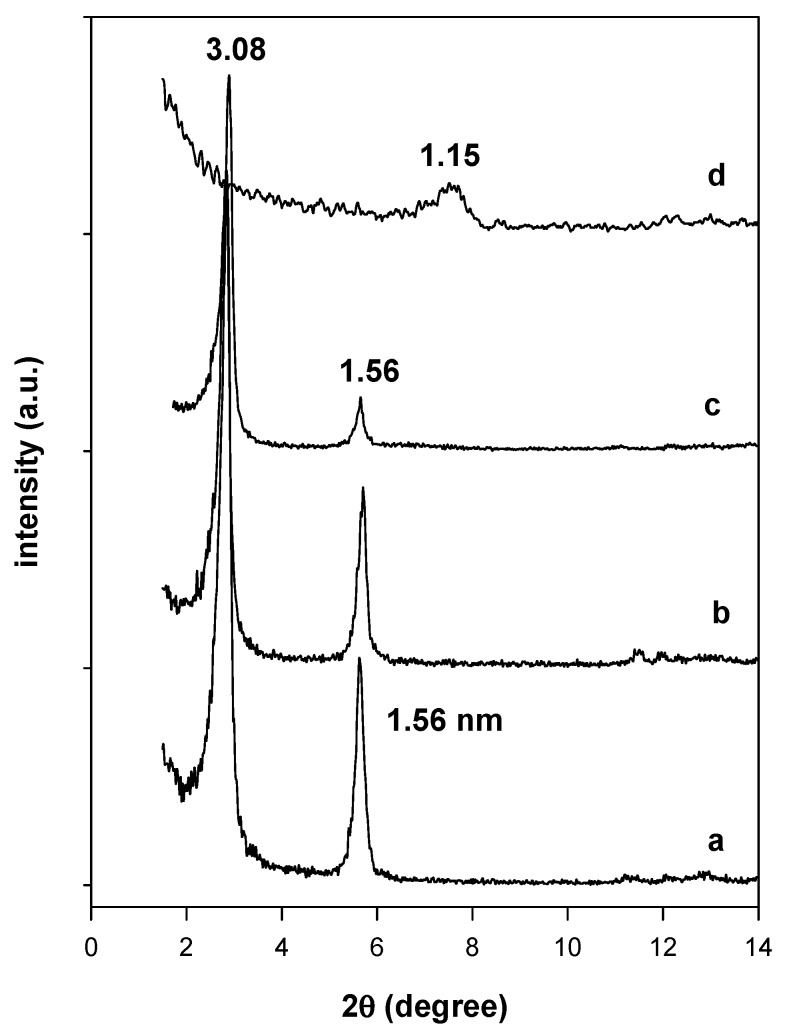
Powder XRD patterns of (**a**) C16Mag-80 treated with different solutions: (**b**) NaOH, (**c**) NaCl, and (**d**) HCl solutions.

The chemical stability tests indicated that the intercalated surfactants were not readily exchanged with Na^+^ cations using either a NaCl solution or a NaOH solution. The powder XRD pattern of the reacted organo-magadiite exhibited a similar pattern with a reflection at 3.08 nm, close to that of the starting organo-magadiite (Figure 4). However, a different powder XRD pattern was obtained when the organo-magadiite was reacted with the HCl solution, exhibiting a reflection at 1.15 nm (Figure 4). This value was close to that reported for H-magadiite in the literature [6,28,42] and indicated that the intercalated C16TMA cations were exchanged with protons from the HCl solution.

The lengths of C16TMA cations in all *trans* configurations were in the range of 2.2 nm to 2.5 nm [28,43]. The basal spacing of Na-magadiite completely dehydrated (similar to H-magadiite) was approximately 1.12 nm [6,28]. The basal spacing was estimated by adding the size of C16TMA cations and the basal spacing of H-magadiite, and had an average of 3.47 nm, higher than the observed value (3.10 nm). The difference between the observed and calculated values was presumably due to some degree of tilting of the vertically C16TMA^+^ cations with the magadiite surface and the presence of H_2_O in the interlayer spacing [44,45]. This configuration was necessary to balance the charge of the silicate layers. The tilt angle in the organo-magadiite was higher than the tilt angle (57° to 59°) in the organo-clays with a lower charge density [27].

### 3.3. Solid-State NMR Studies

The stability of the layered silicate during the exchange process was deduced using solid-state ^29^Si MAS NMR. In fact, the spectrum of the starting Na-magadiite exhibited one main resonance peak centered at −99 mg/L, related to Q^3^-type silicon, and multiple peaks in the range of −110 to −114 mg/L, associated with Q^4^-type sites in the basic layered structure of magadiite (Figure S1) [46]. After the reaction with the C16TMABr solution, a similar spectrum was obtained with main resonance peaks at −99 and −110 mg/L (Figure S1). The overall features of the spectrum did not change with different initial loadings of the surfactant solution, indicating that the layered structure of the silicate material was conserved [5,27].

**Figure 5:**
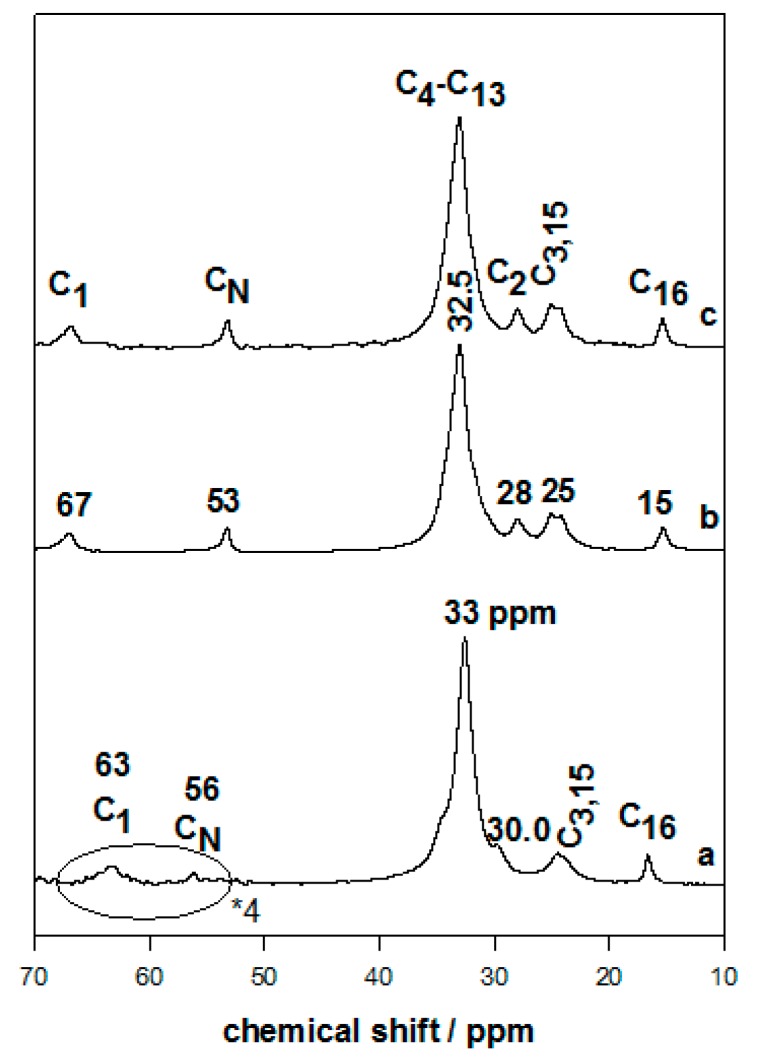
^13^C CP-NMR of (**a**) pure C16TMABr salt, and organo-magadiites exchanged with C16TMABr solution at different concentrations: (**b**) 0.40 mM and (**c**) 0.80 mM.

The presence of the C16TMA cations was confirmed by C, H, N, and XRD techniques, as mentioned previously. The ^13^C CP-NMR technique gave more details about the structure and the conformation of the intercalated cations [47,48]. The ^13^C CP-NMR of the pure C16TMABr solid exhibited an intense resonance peak at 33 mg/L that was assigned to C_4_–C_13_, corresponding to a dominant *trans* conformation for the CH_2_ groups, and an additional shoulder at 30 mg/L associated with a minor degree of the gauche conformation [26,27,36] (Figure 4). The peaks at 63 mg/L and 56 mg/L were related to C_1_ and the methyl groups bonded to N (C_N_), respectively [26,27,36]. Full assignments of the other peaks were described in previous works. The intercalated C16Mag-40 and C16Mag-120 exhibited a resonance spectrum similar to that recorded for the C16TMABr solid with an intense peak at 32.5 mg/L, resulting from the all-*trans* conformation of the organic cations [49] (Figure 5). The conformation was quite homogeneous, which was not the case of other organo-materials with a certain degree of *cis* conformation (with a peak at 30 mg/L). The intensity of the other resonance peaks was enhanced; however, the peaks became broad, and this fact was related to the restricted mobility of the surfactant cations between the silicate layers. In good agreement with the XRD data, the content of the organic cations did not affect the features of the spectra, indicating that the cations mainly adopted the *trans* conformation (Figure 5). Similar data were reported for organo-kenyaites and organo-clays with closely packed organic contents [26,28,36].

### 3.4. Microtextural Studies and Specific Surface Areas

The N_2_ adsorption studies were performed to study the changes in the textural properties of Na-magadiite. The starting Na-magadiite exhibited an adsorption isotherm of type IV, related to a non-porous material, with the condensation of nitrogen molecules occurring at higher relative pressure values and within the voids of the magadiite particles (Figure S2). The organo-magadiites showed the same feature of the N_2_ isotherms with a decrease of the N_2_ uptake at relatively low pressure values. This decrease continued to when the magadiites were fully exchanged with C16TMA cations. The decrease in the N_2_-adsorbed volume indicated that the N_2_ molecules accessed the interlayer space; however, the intercalated C16TMA molecules occupied the active sites, which left the least active sites for N_2_ adsorption. In addition, the BET surface was related to the active sites on the surface, and since the C16TMA intercalation proceeded through cation exchange, the surface adsorption of the intercalated molecules would be accounted for in the S_BET_ surface diminution [36].

The BET surface areas (S_BET_), total pore volume (TPV) and average pore diameter (APD) are summarized in Table 2. The Na-magadiite exhibited a S_BET_ value of 35 m^2^/g, close to that reported for similar materials and other layered silicates such as kanemite and kenyaite [36,39,50]. The modification with C16TMA cations led to a decrease of the S_BET_ value from 23 m^2^/g for the partially exchanged magadiite (C16Mag-20) to 13 m^2^/g for the fully exchanged magadiite (C16Mag-80). These values were similar to those of other organo-silicates and organo-clay minerals [36,51,52]. The average pore volume deduced from the N_2_ isotherms was mainly related to the voids between the particles of the organo-magadiites. In the case of organo-clays, Bhatt et al. have attributed the reduction in the average pore volume to the formation of closely packed aggregates due to interparticle hydrophobic interactions and to the shape of the voids [52]. The APD size increased withthe content of C16TMA^+^ cations (Table 2).

### 3.5. SEM Micrographs

**Figure 6:**
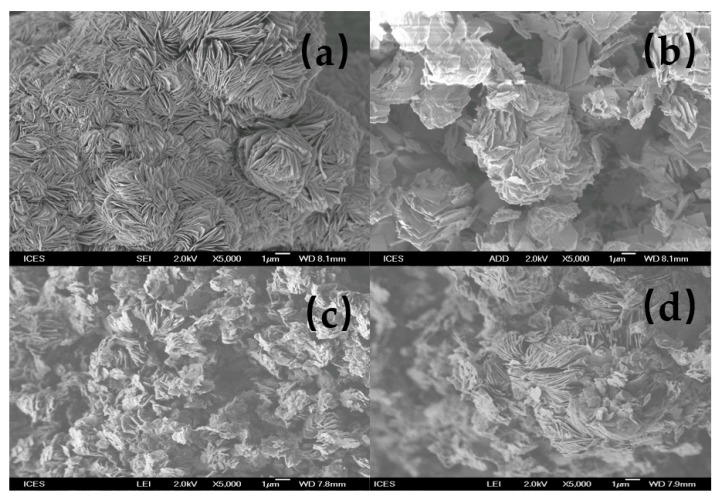
SEM micrographs of (**a**) Na-magadiite treated with different C16TMABr solutions: (**b**) 0.20 mM, (**c**) 0.40 mM, and (**d**) 0.80 mM.

The SEM micrographs of the starting magadiite and the resulting organo-magadiites are presented in Figure 6. Na-magadiite exhibited silicate layers intergrown to form spherical rosettes [38]. When reacted with organic surfactants at initial concentrations of less than 0.8 mM (C16Mag-80), the resulting magadiites exhibited similar morphologies due to the partial exchange. However, for the fully exchanged magadiite, the rosette structure vanished, and the layers of silicates were clearly separated (C16Mag-120). ^29^Si MAS NMR indicated that the layered structure was not altered during the exchange reaction; however, a morphological change occurred. Similar data were reported for Na-kenyaite and Na-magadiite modified by similar cations from a hydroxide solution [26,36].

### 3.6. Thermal Stability

#### 3.6.1. Thermal Gravimetric Analysis (TGA)

**Figure 7:**
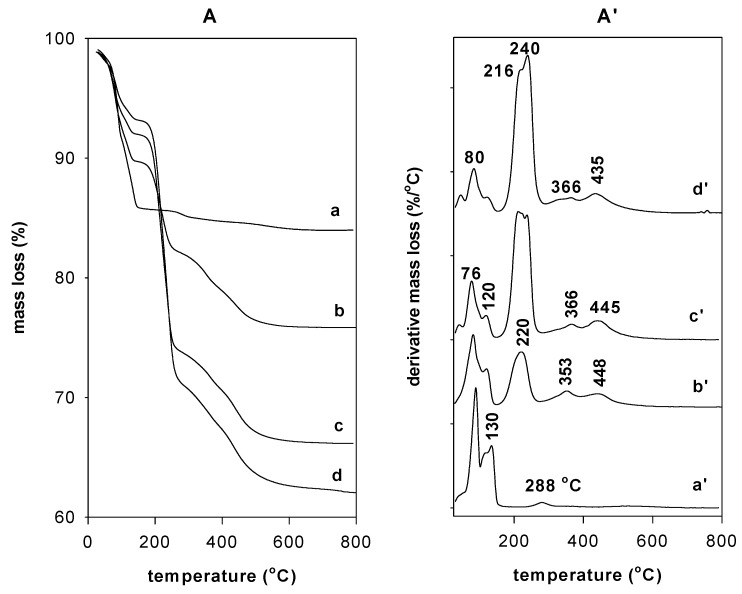
TGA (**A**) and DTG (**A’**) features of (**a**,**a’**) Na-magadiite treated with different concentrations: (**b**,**b’**) 0.40 mM, (**c**,**c’**) 0.80 mM, and (**d**,**d’**) 1.20 mM of C16TMABr solution.

The TGA curve of the as-synthesized Na-Mag showed two general mass losses within the temperature ranges of the respective derivative thermogravimetric analysis (DTG) peak limits. The first mass-loss step of 7.5%, in the range of 26 to 100 °C, corresponded to the loss of the surface-adsorbed water molecules and was associated with a maximum temperature peak at 80 °C. The second mass loss of 7.5%, between 100 to 150 °C, followed the loss of water molecules more strongly bound to Na^+^ cations and was associated with the DTG peak at a maximum temperature of 130 °C. A weak third mass step of 1.2%, at temperatures above 200 °C, was assigned to the dehydroxylation of the silicate layers that occurred at a maximum temperature of 288 °C (Figure 7A). This feature was similar to that reported for the magadiite materials [3,4,39].

After the reaction with the C16TMABr solution with an initial loading of 0.40 mM, the TGA curve exhibited an additional mass-loss step, in the range of 200 °C to 400 °C, that started at 180 °C and was due to the pyrolysis followed the combustion of the organic materials (Figure 7A). This loss was approximately 8% and was accompanied by a maximum loss temperature peak at 220 °C [26]. A continuous mass loss occurred in the temperature range of 290 to 450 °C and was associated with the burnout of the residual carbonaceous material of the decomposed surfactants with two DTG temperatures peaks at 353 and 430 °C [27] (Figure 7A’). As the content of the surfactants increased in the organo-magadiites, the features of the TGA and DTG did not change (Figure 7A,A’); however, the intensity (or the area) of the DTG peak related to the mass loss of the surfactants increased and reached a maximum for organo-magadiite prepared using an initial loading concentration of 2.8 mmol. A mass loss of 28% was achieved in the range of 200 to 350 °C. At the same time, the percentage related to the loss of water molecules decreased, indicating the exchange of Na cations by the organic surfactants and the hydrophobic character of the organo-magadiites and was associated with a decrease in the intensity of the DTG peaks in the temperature range of 25 to 120 °C (Figure 7A’) [36]. Compared to the pure C16TMABr salt (Figure S3), the decomposition of the intercalated surfactants occurred at low temperatures and with different steps, which were associated with the presence of silicate layers that acted as a retardant. 

#### 3.6.2. In Situ Powder XRD Studies

**Figure 8:**
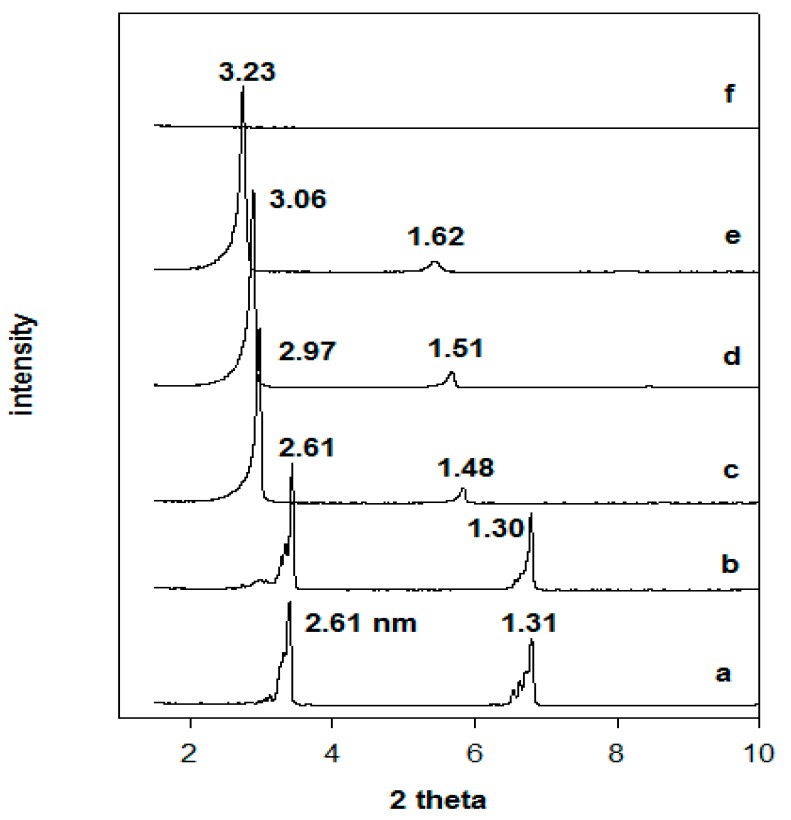
In situ powder XRD patterns of (**a**) C16TMABr solid salt preheated at different temperatures: (**b**) 100 °C, (**c**) 150 °C, (**d**) 200 °C, (**e**) 215 °C, and (**f**) 250 °C.

The thermal stability of the organo-magadiites was followed by in situ studies, meaning the real temperature values were obtained when collecting the powder XRD patterns and without cooling down the samples. To better understand the thermal stability of the organo-magadiites, the thermal stability of the starting magadiite and the C16TMABr salt was studied. In the first stage, the powder XRD pattern of the C16TMABr solid corresponded to a layered structure consisting of a series of (001) reflections with high rationality order at 2.61 nm, 1.31 nm, and 0.86 nm (Figure 8) [27,36]. Indeed, the structure of the C16TMABr salt was described as nonpolar bilayers located between polar layers. The (001) reflection was detected at 2.61 nm, and this value was slightly higher than the length of the C16TMA cations, reported to be close to 2.50 nm [27]. When the C16TMABr salt was preheated at different temperatures, the layered structure was preserved until 215 °C. Then, further expansion of the basal spacing followed, as mainly deduced from the variation of the (001) reflection from 2.61 nm to 3.23 nm. Finally, the C16TMABr melted, and no reflections were detected (Figure 8). The increase in the basal spacing was related to a solid-solid transition phase at 103 °C, as deduced from the differential scanning calorimetry DSC study [53].

**Figure 9:**
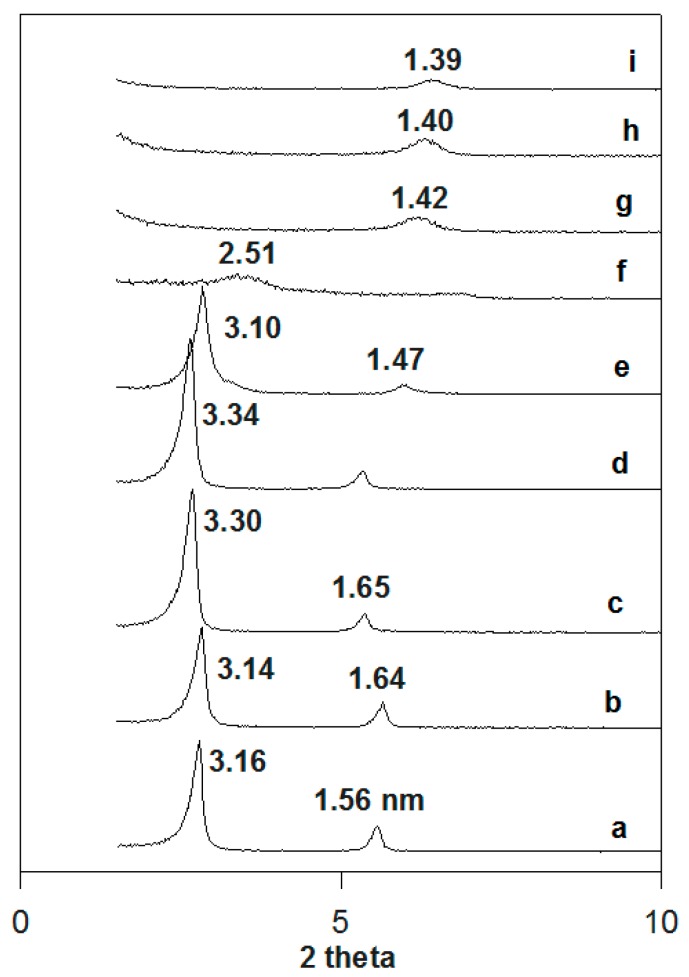
In situ powder XRD patterns of (**a**) C16Mag-80 preheated at different temperatures: (**b**) 50 °C, (**c**) 100 °C, (**d**) 150 °C, (**e**) 200 °C, (**f**) 215 °C, (**g**) 250 °C, (**h**) 300 °C, and (**i**) 400 °C.

For the case of the fully exchanged organo-magadiite (C16Mag-80 was selected as a model sample), the in situ PXRD patterns are depicted in Figure 9. The intercalated C16TMA cations behaved in the same manner as the solid C16TMABr salt. Indeed, the basal spacing of the organo-magadiite increased from 3.16 nm to 3.34 nm when preheated in the temperature range from 50 °C to 150 °C. The reflection at 1.56 nm followed the same behavior, confirming that it was a second-order reflection of the first one. Upon heating at 200 °C, the basal spacing started to shrink to 3.10 nm, and then it collapsed to 2.51 nm at 215 °C. The presence of the silicate layers caused the expansion of the intercalated cations in a short temperature range compared to that of the pure C16TMABr salt. At temperatures higher than 215 °C, further collapse was recorded at 1.42 nm, and the spacing was retained close to this value at higher temperatures, with a slight decrease from 1.42 nm to 1.39 nm. [27]. Loss of crystallinity was observed with a decrease of intensity and general broadening of the XRD reflections.

These obtained values (1.40 nm) were higher than the basal spacings of the pristine Na-magadiite preheated at the same temperature values, as presented in Figure S4. The Na-mag exhibited a basal spacing of 1.15 nm at 200 °C [27]. This fact was related to the presence of residual carbonaceous materials between the silicate layers regenerated during the heating. The loss of water molecules in the interlayer spacing occurred at a temperature of 100 °C, accompanied by a decrease in the basal spacing from 1.54 nm to 1.38 nm and that of water molecules bound to Na cations occurred at a higher temperature of 150 °C, resulting in a further shrinkage of the basal spacing from 1.38 nm to 1.15 nm (Figure S3). These data were in good agreement with the TGA results [39].

### 3.7. Removal of Eosin Studies

#### 3.7.1. Effect of Initial Concentrations (C_i_)

The organo-magadiites were applied as a removal agent for the eosin dye. C16Mag-80 was used as a model sample in this paragraph. A given amount of organo-magadiite can only remove a fixed amount of dye, hence, the initial dye concentration is an important factor to study. Figure S4 shows the variation in the amount removed (milligram of eosin per gram of used material, mg/g) with the initial concentration (C_i_), which varied from 25 mg/L to 1000 mg/L, at an equilibrium time of 18 h and using 0.1 g of material. The increment of initial eosin concentration enhanced the amount of the removed eosin from 2.5 mg/g to 60 mg/g, as the driving force of mass transfer became large [54,55]. In the meantime, the removal percentage was noticed to decrease from 100% at lower C_i_ values (less than 200 mg/L) to 57% at values greater than 700 mg/L (Figure S5). This fact was related to the available surface sites on the organo-magadiite. At lower C_i_ values, sufficient adsorption sites were available for the removal of a smaller number of dye molecules. However, at higher C_i_ values, the number of eosin dye molecules was high compared to the available sites, causing a decrease in the removal efficiency [36]. These data indicated that the organo-magadiite exhibited a performance with the potential to remove eosin from polluted water.

#### 3.7.2. Effect of Organic Content

The removal of eosin was investigated using the organo-magadiites with different organic (surfactant) contents, prepared previously. We previously reported that the organic contents improved the removal capacity of acidic dyes [36,56]. We used magadiite in this fashion due to its higher CEC (200 meq/100 g); thus, a higher organic content on the same order of magnitude as that in organo-clay minerals could be achieved.

**Figure 10:**
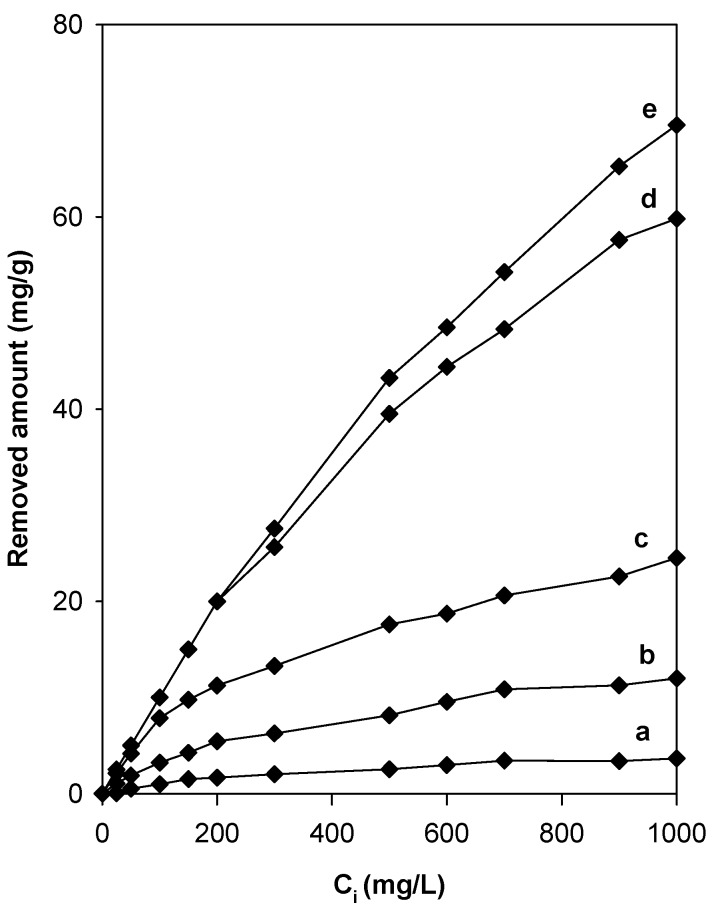
Evolution of the removed amount of eosin with the content of C16TMA^+^ cations in organo-magadiites: (**a**) 0, (**b**) 0.0.17 mmol/g, (**c**) 0.38 mmol/g, (**d**) 0.78 mmol/g, and (**e**) 0.97 mmol/g.

The non-modified Na-magadiite removed an amount of eosin, approximately 4 mg/g (Figure 10), which was lower than those reported for other silicate materials such kenyaite or clay minerals [36,56]. These materials have a consistent negative charge and a poor affinity for negatively charged anionic dyes [57]. Therefore, Na-magadiite had to be modified with suitable quaternary amine cations to enhance its capacity and gain a net positive charge on its surface [58,59]. Figure 10 shows the effect of the modification of Na-magadiite by C16TMA cations on the removal capacity of the eosin dye. For all the tested organo-magadiites, the amount removed was enhanced when the initial concentration (C_i_) was increased from 25 mg/L to 1000 mg/L, and it was highly dependent on the C16TMA content at high initial eosin concentration values above 500 mg/L. The maximum amount of eosin removed was 74 mg/g for C16Mag-120. 

In general, the modification of Na-magadiite with organic surfactants improved the removal properties of eosin compared to that of Na-magadiite, and the organo-magadiites removed a high amount compared to that of the Na-magadiite. Similar results were observed in the case of organo-kenyaites and organo-clays for the removal of eosin or acidic dyes [36,56,58]

The modification of the silicate surface by organic cations and mainly long ones, such as C16TMAs, rendered the magadiite an organophilic material similar to organo-clays [60].The negatively charged surface of the silicate adsorbed the C16TMA^+^ cations via an ion exchange mechanism, where a monolayer of cationic surfactants on the surface of the clay was formed. The positively charged ends of the cationic surfactants were exchanged with the exchangeable interlayer cations of the magadiite (Na^+^), and the hydrophobic head of the cationic surfactants was arranged outward [59]. The C16TMA^+^ cations generated an organophilic phase partition in the interlayer spacing, and the partition occurred through the interaction of the dye with the cationic C16TMA^+^ cations [61,62,63]. 

In other words, the higher removal capacity of eosin by the organo-magadiite was estimated due to the electrostatic attraction between negatively charged of the anionic dye and the positively charged heads of the surfactants. It should be noted that the organo-magadiites exhibited lower specific surface areas; however, the amount of removed eosin remained high, which suggested that the eosin molecules were removed into the interlamellar space. The PXRD patterns after the removal of dyes indicated a slight increase of the basal spacing, and could indicate an anion exchange of Br anions with the removed eosin dyes. 

#### 3.7.3. Effect of Removal Temperature

The removal of eosin was performed at different initial concentrations (C_i_) for a representative sample (C16Mag-80) and at different temperatures. The maximum temperature for the removal properties was selected as 50 °C because no change in the XRD pattern of organo-magadiite was observed (see above paragraph). The data indicated that the removal efficiency at lower initial concentrations was not affected by the temperature change since 100% removal occurred at room temperature (RT) for values from 25 mg/L to 200 mg/L. This fact was due to nearly all the initial eosin being fixed by the available active sites on the adsorbent in accordance with a process that was not temperature dependent. However, at higher initial concentrations above 500 mg/L, the removal percentage and the amount removed depended on the temperature value. At 50 °C, the removal was enhanced to 85% and a maximum of amount removed of 90 mg/g was achieved for a C_i_ of 900 mg/L. These results were due to a higher partitioning rate of dye molecules in organophilic silicates, similar to that of organoclays and organo-kenyaites [36,51], or to the temperature activation of other sites (a function of the intercalated C16TMA) that started to fix eosin molecules and led to the increase of the amount removed. This fact indicated that the removal of eosin was an endothermic process [64].

#### 3.7.4. Effect of Preheated Temperature of Organo-Magadiite

**Figure 11:**
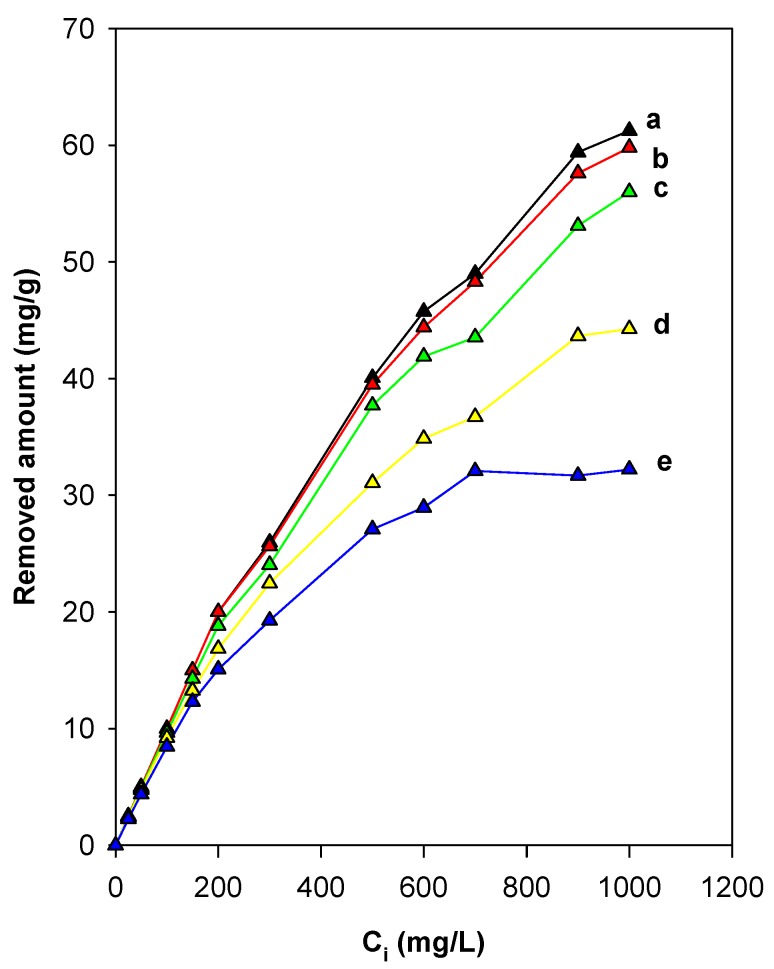
Effect of preheated temperatures of C16Mag-80 on the efficiency of eosin removal: (**a**) RT, (**b**) 100 °C, (**c**) 200 °C, (**d**) 215 °C, and (**e**) 250 °C.

The thermal treatment at a fixed temperature above 200 °C of organo-silicates, such as organo-clays, was used as a method to regenerate spent absorbents for further reuse. In this study, we reported in detail the effect of the preheat treatment of C16Mag-80 on its removal properties. Figure 11 depicts the variation in the amount of removed eosin as a function of the preheated temperature of the organo-magadiite for an initial concentration of 25 to 900 mg/L. The amount removed was not affected by preheated temperatures less than or equal to 150 °C for C_i_ values less than 200 mg/L. A reduction in the amount of eosin removed was noted for organo-magadiite preheated at temperatures higher than 200 °C, and this fact was related to the start of the breakdown of the intercalated C16TMA cations, as indicated by the TGA data and the in situ studies. A noticeable decrease in the eosin amount removed occurred at temperatures greater than 230 °C due to the complete disintegration of the intercalated C16TMA cations (as indicated by the in situ XRD studies), and thus, a loss of the active sites necessary for the removal of eosin.

In comparison to pristine Na-mag, the amount removed was still high, and it could be associated with the presence of the remaining active sites necessary for the removal of the eosin dye, that could have originated from the residual carbonaceous materials. This suggestion would not be useful, as the improvement in the removal capacity was associated with the intercalated C16TMA cations. Nevertheless, the heat treatment may help to find the optimal temperature at which the modified magadiite could be used. Similar data were noticed for an organo-kenyaite preheated at different temperatures for the removal of eosin and for organo-clays for the removal of nitrobenzene [36,65]. 

#### 3.7.5. Maximum Amount of Eosin Removed

To determine the maximum amount of eosin removed using different organo-magadiites under different conditions (before or after the preheat treatment), the Langmuir model was used. This model is based on the assumption that the maximum adsorption corresponds to a saturated monolayer of adsorbate molecules on the adsorbent surface [66]. The linearized Langmuir isotherm allows for the calculation of the adsorption capacity (q_max_) and the Langmuir constant (K_L_) that are equated by the following Equation (1):

(1) $$\;\frac{C_{e}}{q_{e}}=\frac{1}{q_{\max}\;K_{L}}+\frac{C_{e}}{q_{\max}}\;$$

where C_e_ and q_e_ are the concentration at equilibrium (mg/g) and the amount adsorbed at equilibrium (mg/g), respectively, q_max_ is the maximum adsorption capacity (mg/g), and K_L_ is the Langmuir constant (L/mg). These constants can be estimated from the intercept and slope of the linear plot of the experimental data of C_e_/q_e_ versus C_e_.

The isotherms of interest fitted well with this model with a linear square regression correlation coefficient of R^2^ greater than 0.995. The parameters of the Langmuir model are presented in Table 3. 

The maximum amount of eosin removed (q_max_) was enhanced as the content of C16TMA cations increased in the organo-magadiites and reached a maximum of 80.64 mg/g for eosin using the C16Mag-120 sample. Similar data were obtained for organo-kenyaite and clay minerals. However, this value depended on the preheated temperature for a fixed content of C16TMA cations, and a slight variation of the removed capacity was maintained when the sample was preheated at temperatures below 200 °C (before its usage), due to the stability of organo-magadiite in this temperature range (as indicated by the in situ XRD study). However, at temperatures higher than 200 °C, a reduction began, and it continued to decrease with the preheat temperature to a value of 54 mg/g. This fact was due to the initial loss of C16TMA intercalated cations. The increase of K_L_ during the removal of eosin by organo-magadiites could be related to the strong interaction with the silicate surface; however, the role of the C16TMA should also be taken into account, given that the increase was significant in comparison to the K_L_ value for pure Na-magadiite and that for C16Mag-120 preheated at temperatures higher than 215 °C. 

The amount of eosin removed by organo-magadiites was higher than that of organo-kenyaites, local organo-clay minerals and other materials (Table 4). This difference was due to the high organic content on the organophilic magadiite derivatives, which was enhanced by its high cation exchange capacity compared to kenyaite and local clay minerals. However, the organo-magadiites exhibited lower removal capacity compared to ethylenediamine modified chitosan (EDA-CS) and a saccharomyces cerevisiae biosorbent (SC). Nevertheless, the organo-silicates could be considered as a potential candidate for removal of eosin Y dye.

### 3.8. Removal/Regeneration Tests

The removal of dyes from wastewater by solid adsorbents is a transfer process of pollutants from the liquid to the solid phase; thus, it brings another type of pollution for the spent adsorbents because of its disposal in landfills. The regeneration process is of great interest to reuse these spent materials and to reduce further contamination of landfills and its environmental impact. Different methods of regeneration have been investigated including biological, Fenton oxidation, wet air oxidation, microwave, ultrasound, electrochemical, and thermal treatments [74]; each method has its drawbacks, as reported in the literature, mainly involving time consumption and energy use [74].

**Figure 12:**
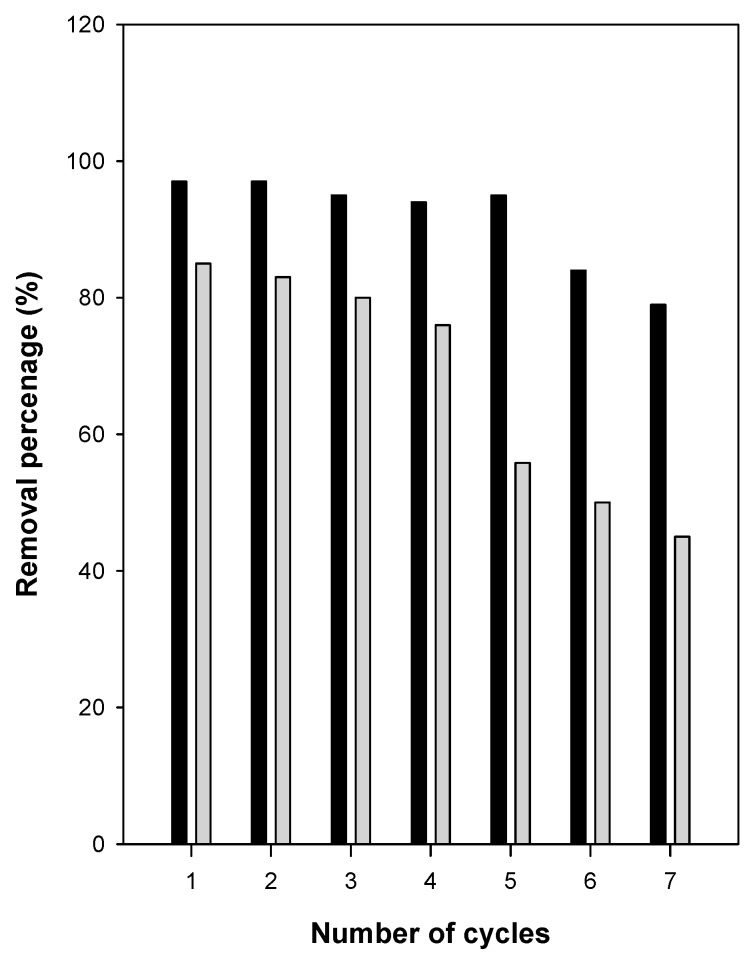
Regeneration/removal cycles of C16Mag-80 using C_i_ of 200 mg/L (dark bars) and 500 mg/L (grey bars), respectively.

In this study, a simple method that did not require many chemicals or a large volume of water was adopted and was used in our previous studies [34,36]. C16Mag-120 was used as the model sample with a C_i_ of 500 mg/L. As shown in Figure 12, the removal efficiency of organo-magadiite was unchanged over three removal/regeneration cycles, and it varied slightly from 86% to 80%. Then, it dropped to 50% after seven cycles. The decrease in the removal efficiency could indicate that some eosin molecules were strongly adhered to the removal sites, making it difficult to remove them. However, using a low C_i_ value of 200 mg/L, the removal efficiency was not altered and remained unchanged for five removal/regeneration cycles. dropping to 80% after the six cycles. These data indicated that organo-magadiite could be used as a potential removal agent for eosin dye.

## 4. Conclusions

The modification of Na-magadiite with C16TMA cations was successfully achieved, and the content of the surfactants could be tuned by using different initial loading concentrations; a maximum C16TMA concentration of 0.97 mmol/g was achieved. The expansion of the basal spacing at 3.08 nm was independent of the intercalated surfactant. The ^13^C CP-NMR study indicated that the intercalated cations exhibited a homogeneous *trans* conformation similar to the C16TMABr solid. The in situ PXRD results indicated an increase in the basal spacing in the temperature range of 50 to 200 °C due to the expansion of the intercalated surfactants. Above this temperature, a decrease in the basal spacing to 2.15 nm occurred and was related to the decomposition of the surfactant cations. 

The removal of negatively charged dye molecules was weak at the Na-magadiite surface; however, the modification with C16TMA exhibited considerable improvement in the removal efficiency and capacity; a maximum of 80.65 mg/g was achieved. This capacity depended on the initial concentration, the operating temperature, and the preheat treatment of C16Mag-120 prior to the removal process. The intercalated cations played an important role in the eosin removal, and their decomposition reduced and affected this property. These results showed that modified magadiite could be used as a potential candidate for the removal of eosin, and its reuse was maintained after three to six cycles of regeneration, depending of the C_i_ values.

## Figures and Tables

**Table 1:** C, H, and N elemental analysis of organo-magadiites prepared with different initial C16TMABr loading solutions.

Sample	C%	H%	N%	Intercalated C16TMA Amount (mmol/g)
C16Mag-20	4.04	1.09	0.06	0.17
C16Mag-40	8.82	2.78	0.21	0.38
C16Mag-80	15.40	3.80	0.73	0.67
C16Mag-120	22.20	4.82	1.11	0.97

**Table 2:** Microtextural properties of Na-magadiite and its organo-derivatives prepared with different initial C16TMABr loading solutions.

Samples	S_BET_ (m^2^/g)	TPV * (cc/g)	APD ^+^ (nm)
Na-mag	35	0.082	10.25
C16Mag-20	23	0.068	11.80
C16Mag-40	20	0.062	12.10
C16Mag-80	15	0.054	13.09
C16Mag-120	13	0.042	12.58

* (TPV) total pore volume, ^+^ (APD) average pore diameter.

**Table 3:** Langmuir parameters for the removal of eosin by different organo-magadiites.

Samples	q_max_ (mgg^−1^)	K_L_ (Lg^−1^)	R^2^
Na-Mag	3.47	0.006	0.931
C16Mag-20	25.06	0.116	0.9943
C16Mag-40	42.54	0.093	0.9961
C16Mag-80	63.06	0.0562	0.9943
C16Mag-120	80.65	0.0712	0.9965
C16Mag-80 (100) *	62.12	0.0562	0.9913
C16Mag-80 (150) *	59.23	0.0551	0.9856
C16Mag-80 (200) *	54.00	0.0284	0.9946
C16Mag-80 (215) *	44.62	0.0216	0.9875
C16Mag-80 (250) *	33.43	0.0207	0.9665

* corresponds to heating temperature value (in °C) of the organo-magadiites.

**Table 4:** Removal capacities of various adsorbents for eosin dye.

Samples	q_max_ (mg/g)	References
Organo-kenyaites	48.01	[36]
Organo-magadiites	63.06–80.65	[This study]
RFA	43.48	[67]
PAC	62.28	[67]
Alumina nanoparticles	47.78	[68]
Organo-local clays	48.66	[56]
Pineapple peels	12.49	[69]
EDA-CS	294.12	[70]
PPy/SD	5.70	[71]
Chitosan hydrobeads	76.00	[72]
SC	200.00	[73]

## Supplementary Materials

Supplementary materials are available online. Figure S1: ^29^Si MAS NMR spectra of (a) Na-magadiite before and after reaction with C16TMABr solution at different initial concentrations (b) 0.20 mM, (c) 0.40 mM, (d) 0.80 mM, Figure S2: Nitrogen adsorption isotherms of (a) Na-magadiite and its organo-derivatives, (b) C16Mag-20, (c) C16Mag-40 and (d) C16Mag-80, Figure S3: TGA (black) and (red) DTG features of C16TMABr salt, Figure S4: in-situ powder XRD patterns of (a) Na-magadiite treated at different temperatures, (b) 50 °C, (c) 100 °C, (d) 150 °C, (e) 200 °C, (f) 250 °C and (g) 400 °C, Figure S5: Removal properties of C16Mag-80 for eosin dye.

## Abbreviation

CEC	Cation exchange capacity
C16TMA	Cetyl trimethylammonium
C16TMAOH	Cetyl trimethylammonium hydroxide
C16TMACl	Cetyl trimethylammonium chloride
C16TMABr	Cetyl trimethylammonium bromide
C16Mag-20	An organo-magadiite with a loaded amount of 20 mM C16TMA cations per 100 g
C16Mag-20	An organo-magadiite with a loaded amount of 40 mM C16TMA cations per 100 g
C16Mag-80	An organo-magadiite with a loaded amount of 80 mM C16TMA cations per 100 g
C16Mag-120	An organo-magadiite with a loaded amount of 120 mM C16TMA cations per 100 g
UV	UV visible
Co(NO_3_)_2_·6H_2_O	Cobalt nitrate hexahydrate
XRD	X-ray diffraction
SEM	Scanning electron microscope
NMR	Nuclear magnetic resonance
CP	Coupled proton
MAS	Magic Angle Spinning
EDX	Energy dispersive X-ray spectroscopy
T.P.V.	Total pore volume
A.P.D.	Average pore diameter
TGA	Thermal gravimetric Analysis
DTG	Derivative thermal gravimetric
DSC	Differential scanning calorimetry
q_max_	Maximum adsorption capacity (mg/g)
K_L_	Langmuir constant (L/mg)
C_i_	Initial concentration
RFA	Raw fly carbon ash
PAC	Powdered activated carbon
PPy/SD	Polymerpyrrole/sawdust
EDA-CS	Ethylenediamine modified chitosan
SC	Saccharomyces cerevisiae biosorbent

## References

[1] Selvam T., Inayat A., Schwieger W. (2014). Reactivity and applications of layered silicates and layered double hydroxides. Dalton Trans..

[2] Kooli F., Mianhui L., Plevert J. (2006). Comparative studies on the synthesis of Na-magadiite, Na-kenyaite and RUB-18 phases. Clay Sci..

[3] Lagaly G., Beneke K., Weiss A. (1975). Magadiite and H-magadiite: I. Sodium magadiite and some of its derivatives. Am. Mineral..

[4] Mokhtar A., Djelad A., Adjdir M., Zahraoui M., Bengueddach A., Sassi M. (2018). Intercalation of hydrophilic antibiotic into the interlayer space of the layered silicate magadiite. J. Mol. Struct..

[5] Wang S.F., Lin M.L., Shieh Y.N., Wang Y.R., Wang S. (2007). Organic modification of synthesized clay-magadiite. Ceram. Int..

[6] Macedo T.R., Petrucelli G.C., Airoldi C. (2007). Silicic acid magadiite as a host for *N*-alkyldiamine guest molecules and features related to the thermodynamics of intercalation. Clays Clay Miner..

[7] Paz G.L.E., Munsignatti C.O., Pastore H.O. (2016). Novel catalyst with layered structure: Metal substituted magadiite. J. Mol. Catal. A Chem..

[8] Vieira R.B., Moura P.A.S., Vilarrasa-Garcia E., Azevedo D.C.S., Pastore H.O. (2017). Polyamine-Grafted Magadiite: High CO_2_ Selectivity at Capture from CO_2_/N_2_ and CO_2_/CH_4_ Mixtures. J. CO_2_ Util..

[9] Zhang Y.F., Wang Q.S., Gao S.N., Jiang H.M., Meng C.G. (2018). Intercalation and in situ formation of coordination compounds with ligand 8-hydroxyquinoline-5-sulfonic acid in the interlayer space of layered silicate magadiite by solid-solid reactions. Microp. Mesop. Mater..

[10] Yufeng C., Bao Y., Yan Z. (2016). Intercalation of Tb into magadiite and characterization of Tb-intercalated magadiites. Clay Miner..

[11] Mokhtar A., Djelad A., Boudia A., Sassi M., Bengueddach A. (2017). Preparation and characterization of layered silicate magadiite intercalated by Cu^2+^ and Zn^2+^ for antibacterial behavior. J. Porous Mater..

[12] Kooli F., Kiyozumi Y., Mizukami F. (2001). Novel Layered silicate and microporous silica materials in the Na-magadiite-H_2_O-(TMA)_2_O system. New J. Chem..

[13] Lv T.M., Zhang S.L., Feng Z., Wang F.S., Zhang S.Q., Zheng J.Q., Liu X., Meng C.G., Wang Y. (2017). Synthesis of zeolite omega by the magadiite conversion method and insight into the changes of medium-range structure during crystallization. Cryst. Growth Des..

[14] Pires C.T., Oliveira N.G., Airoldi C. (2012). Structural incorporation of titanium and/or aluminum in layered silicate magadiite through direct synthesis. Mater. Chem. Phys..

[15] Novodarszki G., Valyon J., Illes A., Dobe S., Mihalyi M.R. (2017). Synthesis and characterization of Al-magadiite and its catalytic behavior in 1,4-pentanediol dehydration. React. Kinet. Mech. Catal..

[16] Maeno Z., Mitsudomem T., Mizugaki T., Jitsukawa K., Kaneda K. (2015). Selective C–C Coupling Reaction of Dimethylphenol to Tetramethyldiphenoquinone Using Molecular Oxygen Catalyzed by Cu Complexes Immobilized in Nanospaces of Structurally-Ordered Materials. Molecules.

[17] Zebib B., Lambert J.F., Blanchard J., Breysse M. (2006). LRS-1: A New Delaminated Phyllosilicate Material with High Acidity. Chem. Mater..

[18] Moura H.M., Bonk F.A., Pastore H.O. (2012). Pillaring cetyltrimethylammonium-magadiite: A stepwise method to mesoporous materials with controlled pores sizes and distribution. Eur. J. Miner..

[19] Ma J., Cui B., Li D. (2011). Mechanism of adsorption of anionic dye from aqueous solutions onto organobentonite. J. Hazard. Mater..

[20] Middea A., Spinelli L.S., Souza F.G., Neumann R., Fernandes T.L.A.P., Gomes O.F.M. (2017). Preparation and characterization of an organo-palygorskite-Fe_3_O_4_ nanomaterial for removal of anionic dyes from wastewater. Appl. Clay Sci..

[21] Ghavami M., Zhao Q., Javadi S., Jangam J.S.D., Jasinski J.B., Saraei N. (2017). Change of organobentonite interlayer microstructure induced by sorption of aromatic and petroleum hydrocarbons—A combined study of laboratory characterization and molecular dynamics simulations. Colloids Surf. A.

[22] Wang G., Wang S., Sun Z., Zheng S., Xi Y. (2017). Structures of nonionic surfactant modified montmorillonites and their enhanced adsorption capacities towards a cationic organic dye. Appl. Clay. Sci..

[23] Hamoudi S., Yang Y., Moudrakovski I., Lang S., Sayari A. (2001). Synthesis of Porous Organosilicates in the Presence of Alkytrimethylammonium Chlorides: Effect of the Alkyl Chain Length. J. Phys. Chem. B.

[24] He H., Ma L., Zhu J., Frost R.L., Theng B.K.G., Bergaya F. (2014). Synthesis of organoclays: A critical review and some unresolved issues. Appl. Clay Sci..

[25] Kooli F., Qin L.S., Kiat Y.Y., Weirong Q., Hian P.C. (2006). Effect of hexadecyltrimethylammonium (C16TMA) counteranions on the intercalation properties of different montmorillonites. Clay Sci..

[26] Kooli F., Khimyak Y.Z., Alshahateet S.F., Chen F. (2005). Effect of the acid activation levels of montmorillonite clay on the cetyltrimethylammonium cations adsorption. Langmuir.

[27] Kooli F., Mianhui L., Alshahateet S.F., Fengxi C., Zhu Y. (2006). Characterization and thermal stability properties of intercalated Na-magadiite with cetyltrimethylammonium (C16TMA) surfactants. J. Phys. Chem. Solids.

[28] Kooli F., Yan L. (2009). Thermal stable cetyl trimethylammonium-magadiites: Influence of the surfactant solution type. J. Phys. Chem. C.

[29] Royer B., Natali F.C., Lima E.C., Macedo T.R., Airoldi C. (2010). Sodic and Acidic Crystalline Lamellar Magadiite Adsorbents for the Removal of Methylene Blue from Aqueous Solutions: Kinetic and Equilibrium Studies. Sep. Sci. Technol..

[30] Royer N.F., Cardoso E.C., Lima T.R., Macedo C., Airoldi A. (2010). Useful organofunctionalized layered silicate for textile dye removal. J. Hazard. Mater..

[31] Guerra D.L., Pinto A.A.J., Souza A., Airoldi C., Viana R.R. (2009). Kinetic and thermodynamic uranyl (II) adsorption process into modified Na-Magadiite and Na-Kanemite. J. Hazard. Mater..

[32] Mokhtar M. (2017). Application of synthetic layered sodium silicate magadiite nanosheets for environmental remediation of methylene blue dye in water. Materials.

[33] Cooksey C.J. (2018). Quirks of dye nomenclature. 10. Eosin Y and its close relatives. Biotech. Histochem..

[34] Kooli F., Liu Y., Al-Faze R., Al-Suhaimi A. (2015). Effect of acid activation of Saudi local clay mineral on removal properties of basic blue 41 from an aqueous solution. Appl. Clay Sci..

[35] Ramos-Vianna M.M.G., Dweck J., Kozievitch F.J., Valenzuela-Diaz F.R., Buchler P.M. (2005). Characterization and study of sorptive properties of differently prepared organoclays from a Brazilian natural bentonite. J. Therm. Anal. Calorim..

[36] Kooli F., Liu Y., Hbaieb K., Al-Faze R. (2018). Characterization of organo-kenyaites: Thermal stability and their effects on eosin removal characteristics. Clay Miner..

[37] Yukutake H., Kobayashi M., Otsuka H., Takahara A. (2009). Thermal Degradation Behavior of Polystyrene/Magadiite Nanocomposites Prepared by Surface-initiated Nitroxide-Mediated Radical Polymerization. Polym. J..

[38] Wang Y.R., Wang S.F., Chang L.C. (2006). Hydrothermal synthesis of magadiite. Appl. Clay Sci..

[39] Moura A.O., Prado A.G. (2009). Effect of thermal dehydration and rehydration on Na-magadiite structure. J. Colloid Interface Sci..

[40] Wang D., Jiang D.D., Pabst J., Han Z., Wang J., Wilkie C.A. (2004). Polystyrene magadiite nanocomposites. Polym. Eng. Sci..

[41] Kooli F. (2009). Exfoliation Properties of acid-activated montmorillonites and their resulting organoclays. Langmuir.

[42] Asakura Y., Hosaka N., Osada S., Terasawa T., Shimojima A., Kuroda K. (2015). Interlayer Condensation of Protonated Layered Silicate Magadiite through Refluxing in *N*-Methylformamide. Bull. Chem. Soc. Jpn..

[43] Vidal N., Volzone C. (2012). Influence of organobentonite structure on toluene adsorption from water solution. Mater. Res..

[44] Peng S., Gao Q., Wang Q., Shi J. (2004). Layered Structural Heme Protein Magadiite Nanocomposites with High Enzyme-like Peroxidase Activity. Chem. Mater..

[45] Zhu L., Zhu R. (2008). Surface structure of CTMA^+^ modified bentonite and their sportive characteristics towards organic compounds. Colloids Surf. A.

[46] Thiesen P.H., Beneke K., Lagaly G. (2002). Silylation of a crystalline silicic acid: An MAS NMR and porosity study. J. Mater. Chem..

[47] Wang L.Q., Liu J., Exarhos G.J., Flanigan K.Y., Bordia R. (2000). Conformation Heterogeneity and Mobility of Surfactant Molecules in Intercalated Clay Minerals Studied by Solid-State NMR. J. Phys. Chem. B.

[48] Gerstmans A., Urbanczyk L., Jérôme R., Robert J.L., Grandjean J. (2008). XRD and NMR characterization of synthetic hectorites and the corresponding surfactant-exchanged clays. Clay Clays Miner..

[49] He H., Frost R.L., Deng F., Zhu J., Wen X., Yuan P. (2004). Conformation of surfactant molecules in the interlayer of montmorillonite studied by ^13^C MAS NMR. Clays Clay Miner..

[50] Bi Y., Lambert J.F., Millot Y., Casale S., Blanchard J., Zeng S., Nie H., Li D. (2011). Relevant parameters for obtaining high-surface area materials by delamination of magadiite, a layered sodium silicate. J. Mater. Chem..

[51] Bhatt A.S., Sakaria P.L., Vasudevan M., Pawar R.R., Sudheesh N., Bajaj H.C., Mody H.M. (2012). Adsorption of an anionic dye from aqueous medium by organoclays: Equilibrium modeling, kinetic and thermodynamic exploration. RSC Adv..

[52] Baskaralingam P., Pulikesi M., Elango D., Ramamurthi V., Sivanesan S. (2006). Adsorption of acid dye onto organobentonite. J. Hazard. Mater..

[53] Bezrodna T., Puchkovska G., Styopkin V., Baran J., Drozd M., Danchuk V., Kravchuk V. (2010). IR-study of thermotropic phase transitions in cetyltrimethylammonium bromide powder and film. J. Mol. Struct..

[54] Atar N., Olgun A., Colak F. (2008). Thermodynamic, Equilibrium and Kinetic Study of the Biosorption of Basic Blue 41 using Bacillus maceran. Eng. Life Sci..

[55] Mall I.D., Srivastava V.C., Agarwal N.K., Mishra I.M. (2005). Removal of congo red from aqueous solution by bagasse fly ash and activated carbon: Kinetic study and equilibrium isotherm analyses. Chemosphere.

[56] Al-Faze R., Kooli F. (2014). Eosin removal properties of organo-local clay from aqueous solution. Orient. J. Chem..

[57] Crini G. (2006). Non-conventional low-cost adsorbents for dye removal: A review. Bioresour. Technol..

[58] Özcan A., Öncü E.M., Özcan A.S. (2004). Adsorption of Acid Blue 193 from aqueous solutions onto Na-bentonite and DTMA-bentonite. J. Colloid Interface Sci..

[59] Ahmadishoar J., Bahrami S.H., Movassagh B., Amirshahi S.H., Arami M. (2017). Removal of disperse blue 56 and disperse red 135 dyes from aqueous dispersions by modified montmorillonite nanoclay. Chem. Ind. Chem. Eng. Q.

[60] Park Y., Ayoko G., Frost R.L. (2011). Characterisation of Organoclays and Adsorption of p-Nitrophenol: Environmental Application. J. Colloid Interface Sci..

[61] Jovic-Jovicic N., Milutinovic-Nikolic A., Grzetic I., Jovanovic D. (2008). Organobentonite as efficient textile dye sorbent. Chem. Eng. Technol..

[62] Lee S.Y., Kim S.J. (2002). Adsorption of naphthalene by HDTMA modified kaolinite and halloysite. Appl. Clay Sci..

[63] Onal M., Sarikaya Y. (2007). Some physicochemical properties of partition nanophase formed in sportive organoclays. Colloids Surf. A.

[64] Hattacharyya K.G., Sarma A. (2003). Adsorption characteristics of the dye, brilliant green, on neem leaf powder. Dyes Pigment..

[65] Borisover M., Bukhanovsky N., Lapides I., Yariv. S. (2010). Mild pre-heating of organic cation-exchanged clays enhances their interactions with nitrobenzene in aqueous environment. Adsorption.

[66] Langmuir I. (1916). The constitution and fundamental properties of solids and liquids. J. Am. Chem. Soc..

[67] Bello O.S., Olusegun O.A., Njoku V.O. (2013). Fly ash; an alternative to powdered activated carbon for the removal of eosin dye from aqueous solutions. Bull. Chem. Soc. Ethiop..

[68] Thabet M.S., Ismaiel A.M. (2016). Sol-gel γ-Al2O3 nanoparticles assessment of the removal of eosin yellow using: Adsorption, kinetic and thermodynamic parameters. J. Encapsulation Adsorpt. Sci..

[69] Ugbe F.A., Ikudayisi V.A. (2017). The kinetics of eosin yellow removal from aqueous solution using pineapple peels. Edorium J. Waste Manag..

[70] Huang X.Y., Bin J.P., Bu H.T., Jiang G.B., Zeng M.H. (2011). Removal of anionic dye eosin Y from aqueous solution usingethylenediamine modified chitosan. Carbohydr. Polym..

[71] Ansari R., Mosayebzadeh Z. (2010). Removal of Eosin Y, an anionic dye, from aqueous solutions using conducting electroactive polymers. Iran. Polym. J..

[72] Chatterjee S.S., Chatterjee B.P., Das A.R., Guha A.R. (2005). Adsorption of a model anionic dye, eosin Y, from aqueous solution by chitosan hydrobeads. J. Colloid Interface Sci..

[73] Bahramifar N., Tavasolli M., Younesi H. (2015). Removal of eosin Y and eosin B dyes from polluted water through biosorption using Saccharomyces cerevisiae: Isotherm, kinetic and thermodynamic studies. J. Appl. Res. Water Wastewater.

[74] Shahadat M.M., Isamil S. (2018). Regeneration performance of clay-based adsorbents for the removal of industrial dyes: A review. RSC Adv..

