# Supplementary material for: Application of Organo-Magadiites for the Removal of Eosin Dye from Aqueous Solutions: Thermal Treatment and Regeneration
Source: Molecules. 2018 Sep 6;23(9):2280. doi: 10.3390/molecules23092280 (PMC6225231; doi:10.3390/molecules23092280)
Supplement: Supplementary file 1 [file molecules-23-02280-s001.pdf]

Supporting Information

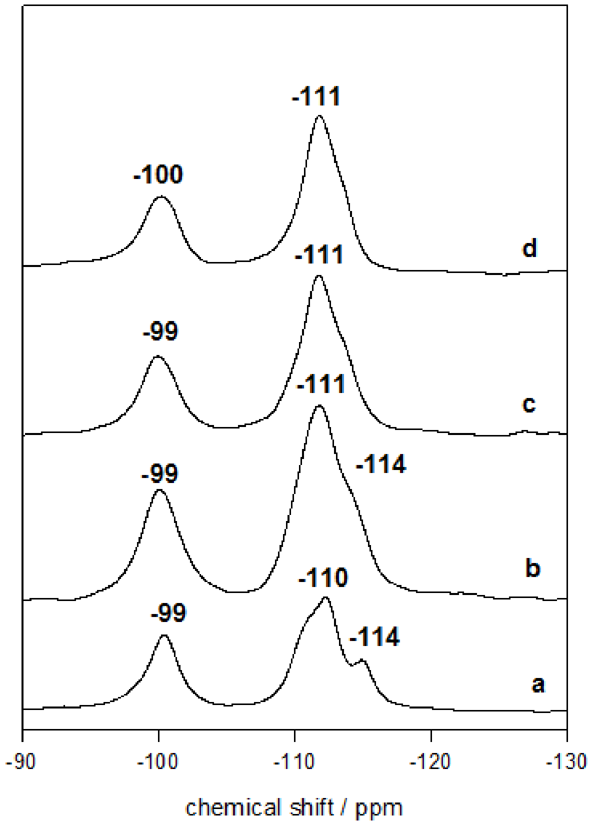

Figure S1. ^{29}Si MAS NMR spectra of (**a**) Na-magadiite before and after reaction with C16TMABr solution at different initial concentrations (**b**) 0.20 mM, (**c**) 0.40 mM, and (**d**) 0.80 mM.

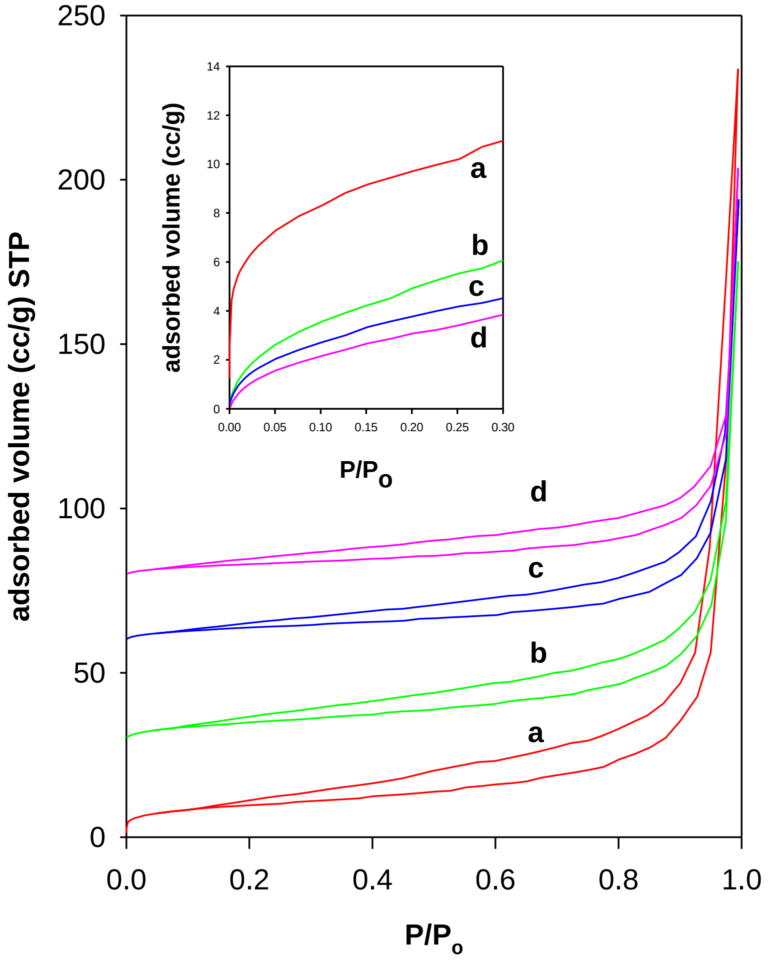

Figure S2. Nitrogen adsorption isotherms of (**a**) Na-magadiite and its organo-derivatives, (**b**) C16Mag-20, (**c**) C16Mag-40, and (**d**) C16Mag-80.

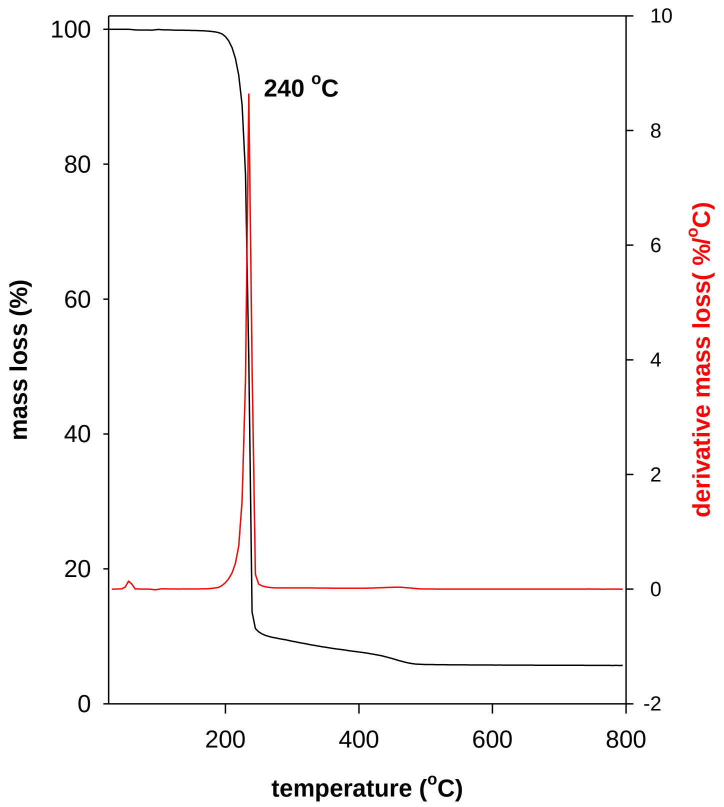

Figure S3. TGA (black) and (red) DTG features of C16TMABr salt.

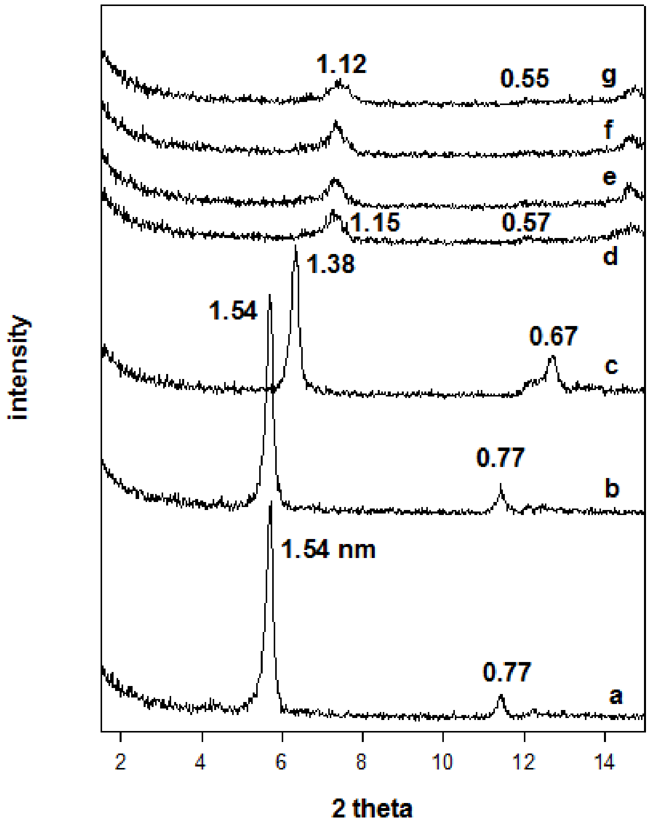

Figure S4. in-situ powder XRD patterns of (**a**) Na-magadiite treated at different temperatures, (**b**) 50 °C, (**c**) 100 °C, (**d**) 150 °C, (**e**) 200 °C, (**f**) 250 °C, and (**g**) 400 °C.

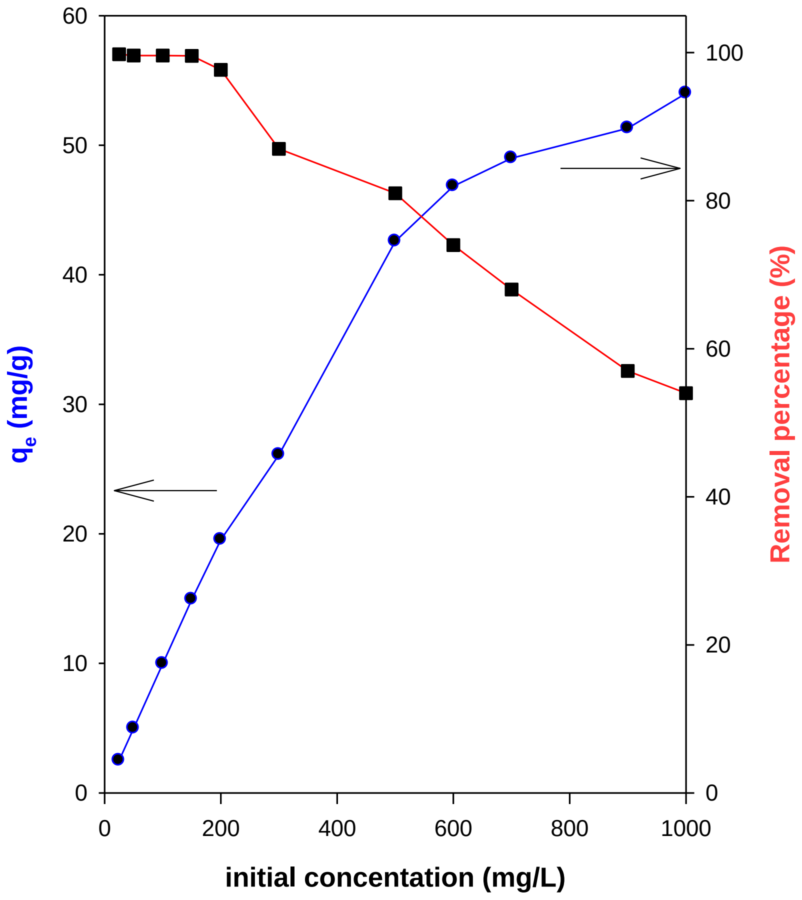

Figure S5. Removal properties of C16Mag-80 for eosin dye.
